# In Vitro Assessment of Chitosan‐PEG Hydrogels Enriched with MSCs‐Exosomes for Enhancing Wound Healing

**DOI:** 10.1002/mabi.202400609

**Published:** 2025-01-21

**Authors:** Masoumeh Ezati, Amir Hashemi, Inna Zumberg, Minoo Partovi Nasr, Zdenka Fohlerova

**Affiliations:** ^1^ Department of Biomedical Engineering Faculty of Electrical Engineering and Communication Brno University of Technology Technicka 3082/12 Brno 61600 Czech Republic; ^2^ Department of Microelectronics Faculty of Electrical Engineering and Communication Brno University of Technology Technicka 3082/12 Brno 61600 Czech Republic

**Keywords:** angiogenesis, cell migration, chitosan‐polyethylene glycol hydrogel, mesenchymal stem cells derived exosomes, wound healing

## Abstract

Regenerating skin tissue remains a major challenge in medical science, especially due to the risk of scarring and prolonged healing, which becomes even more complicated in people with diabetes. Recent advancements have led to the creation of therapeutic dressings incorporating drug‐delivery systems to tackle these issues. Exosomes (Exos) derived from mesenchymal stem cells (MSCs) have gained significant attention for mediating therapy without directly using cells, thanks to their natural anti‐inflammatory and tissue repair properties mirroring those of MSCs. In this study, an advanced wound dressing combines chitosan (CS) and polyethylene glycol (PEG) hydrogel with adipose MSCs‐derived Exos (ADMSCs‐Exos). This composite, formed using a straightforward blending technique, is engineered to improve the healing process of severe skin injuries by steadily releasing Exos as the hydrogel degrades. The in vitro studies demonstrate that this hydrogel‐exosome dressing greatly enhances endothelial cell migration, reduces oxidative stress, and promotes angiogenesis, crucial for effective wound healing. Additionally, real time‐polymerase chain reaction (RT‐PCR) analysis revealed significant upregulation of key genes involved in these processes, supporting the therapeutic potential of the hydrogel‐Exo combination. These findings emphasize the potential of this hydrogel‐Exos combination as an innovative and promising solution for advanced wound care.

## Graphical abstract

A novel hydrogel composed of chitosan and polyethylene glycol, integrated with exosomes derived from adipose mesenchymal stem cells, demonstrates enhanced wound healing. This biomaterial promotes endothelial cell migration, reduces oxidative stress, and facilitates angiogenesis. These findings reveal a promising strategy for advanced wound care, offering sustained exosome release for effective tissue repair.

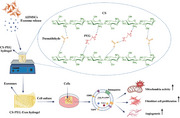

## 1 Introduction

Healing skin injuries cont*i*nues to be a serious therapeutic challenge, causing substantial financial strain on patients. Skin wound healing involves a sequence of intricate biological processes such as hemostasis, inflammation, proliferation, and tissue remodeling.^[^ ^1^ ^]^ Rapid wound healing and scar‐free regeneration are crucial for successful skin restoration. Conventional methods have mostly depended on physical dressings to protect against microbial invasion but have been inadequate in promoting significant tissue regeneration.^[^ ^2^ ^]^

In the field of regenerative medicine, cell‐based therapies, namely those utilizing MSCs, have garnered considerable attention for their ability to aid in skin regeneration. MSCs are multipotent cells derived from mesodermal and ectodermal layers present in tissues such as bone marrow, adipose tissue, and umbilical cord. They are known for their ability to modulate the immune system, release signaling molecules, respond to chemical signals, and aid in tissue repair. Recent findings indicate that the therapeutic effectiveness of MSCs mostly comes from the substances they secrete, rather than the cells themselves.^[^ ^3^ ^]^

Exos are a type of extracellular vesicles with a bilayer membrane that contain biomolecules like mRNA, miRNA, and proteins. They are important for cell communication and reflect the activity of the cells they come from.^[^ ^4^ ^]^ MSCs‐Exos mimic the positive characteristics of MSCs, such as anti‐inflammatory effects, prevention of fibrosis, and support for cell survival, differentiation, and blood vessel formation, making them promising cell‐free treatment options.^[^ ^5^ ^]^ Among the various sources, ADMSCs‐Exos have gained prominence in skin wound healing studies. ADMSCs‐Exos have shown the capacity to improve fibroblast movement and growth in a lab setting and trigger the Wnt/β‐catenin signaling pathway, which is important for healing wounds.^[^ ^6^ ^]^ However, the translation of Exos‐based therapeutics to practical use is impeded by the quick removal and brief duration of Exos in the body.^[^ ^7^ ^]^ ADMSCs‐Exos are being incorporated into scaffold materials to distribute them specifically to wound locations, leading to the study of different hydrogel formulations. CS‐based hydrogels are well‐known for their biocompatibility, biodegradability, and natural antibacterial characteristics, making them very useful for wound healing. CS, derived from chitin, facilitates the creation of hydrogels by physical and chemical cross‐linking processes, promoting a wet environment that supports tissue healing. CS hydrogels have drawbacks including poor mechanical strength, uncontrolled swelling, and quick disintegration, which may hinder their use as wound dressings or scaffolds for tissue regeneration.^[^ ^8^ ^]^

To overcome these limitations and enhance the hydrogel's function, PEG was incorporated to improve their structural integrity and performance. PEG, a synthetic polymer that is hydrophilic and biocompatible, is praised for its capability to improve the mechanical strength, flexibility, and hydration ability of hydrogels. PEG included into the CS matrix regulates the hydrogel's degradation rate and swelling properties, creating a stable system that promotes optimal wound healing conditions without excessive breakdown.^[^ ^9^ ^]^ This balance ensures that the hydrogel remains at the wound site for an extended period, reducing the necessity for frequent dressing changes and improving patient comfort.^[^ ^10^ ^]^ Additionally, PEGylation reduces the strong bioadhesive characteristics of CS, making it easier to remove the dressing without harming the newly developed tissue. This trait is especially important in delicate wound care situations, aiming to reduce patient suffering and avoid any interference with the healing process.^[^ ^11^ ^]^ Moreover, PEG's capacity to decrease protein adsorption and cellular adherence to the hydrogel helps limit immunogenic reactions and prolongs the hydrogel's presence at the wound site, enhancing the controlled release of therapeutic substances such as Exos.^[^ ^12^ ^]^

This study involved the synthesis of a novel hydrogel by blending CS with PEG, and uniquely integrates ADMSCs‐Exos CS‐PEG hydrogels into this biocompatible matrix, creating a multifunctional hydrogel with sustained therapeutic effects. The original design of a system allows controlled release of Exos over 14 days, ensuring continuous bioactivity at the wound site. This extended release addresses a common limitation of Exos‐based therapies, which are often hindered by their rapid clearance. Additionally, this study is among the first to investigate the hydrogel's ability to mitigate oxidative stress and preserve mitochondrial integrity, two critical factors in efficient wound healing. We conducted a series of in vitro experiments to evaluate the effects of the hydrogel on cell viability, migration, and angiogenesis. In addition, we analyzed the levels of oxidative stress and the morphology of mitochondria to gain insights into how the hydrogel could potentially mitigate oxidative damage and promote cellular health in wound healing. In addition, a gene expression analysis using qRT‐PCR was performed to examine the specific molecular mechanisms that are responsible for these effects. The analysis specifically focused on genes associated with angiogenesis and inflammation. The thorough evaluations may emphasize the hydrogel's potential as a cutting‐edge remedy for enhancing the healing process of skin wounds.

## 2 Experimental Section

### 2.1 Materials

Chitosan (medium molecular weight), polyethylene glycol (PEG), fetal bovine serum (FBS), acetic acid, sodium hydroxide (NaOH), phosphate buffered saline (PBS), acetone, paraformaldehyde, ammonium persulfate (APS), hydrochloric acid (HCl), uranyl acetate, dimethyl sulfoxide (DMSO), DAPI (4′,6‐diamidin‐2‐fenylindol), penicillin/streptomycin (PS), norepinephrine, L‐glutamine, Dulbecco's Modified Eagle Medium (DMEM), DiI (1,1′‐Dioctadecyl‐3,3,3′,3′‐tetramethylindocarbocyanine perchlorate), and Thiazolyl Blue Tetrazolium Bromide were purchased from Sigma‐Aldrich. Pierce BCA Protein Assay Kit, CD63 Monoclonal Antibody, HRP‐conjugated secondary antibodies, cDNA Synthesis Kit, and ActinGreen 488 dye were obtained from Thermo Fisher Scientific. ECGM‐MG endothelial cell growth medium was obtained from Cell Lines Service (CLS). RNeasy Mini Kit was purchased from Qiagen (Hilden, Germany). LightCycler 480 High Resolution Melting Master SYBR Green amplification kit was purchased from Roche (Basel, Switzerland), and Ham‘s F12 w/ L‐Glutamine medium (MCL‐029‐500ML) was purchased from Serana Europe GmbH (Brandenburg, Germany).

### 2.2 Cell Culture

ADMSCs were grown in DMEM supplemented with 10% FBS and 1% PS and kept at 37 °C in a 5% CO_2_ environment for this study. The culture media were renewed every two days. During biological experiments, the cells were trypsinized, suspended in fresh culture fluid, and used for the intended research. Cells at passages 3 to 4 were chosen for all experimental protocols. NIH/3T3 mouse fibroblast cells (ATCC, USA) were cultured in DMEM high glucose and Ham‘s F12 w/ L‐Glutamine (50/50) with 10% FBS, and 1% PS. The samples were cultured at 37 °C in a humidified atmosphere containing 5% CO_2_. Human umbilical vein endothelial cells (HUVECs) were cultured in ECGM‐MG complete medium, which was supplemented with 1% PS.

### 2.3 Exo Isolation and Characterization

ADMSCs were grown in cell culture flasks until they reached 80% confluency. Then, the media was changed to DMEM without FBS to guarantee that the separated exosomes came only from the specific cells. This was done since regular FBS has elevated quantities of exosomes that may adulterate the exosomes obtained from the cells. Exosomes were isolated from pooled conditioned media obtained after cultivating the cells for 48 h in serum‐free conditions. The removal of cellular debris was achieved using centrifugation at 300 g for 10 min, followed by a centrifugation at 10,000 g for 30 min to eliminate microvesicles. The conditioned media underwent ultracentrifugation at 100,000 g at 4 °C for 120 min. The supernatant was removed, and the exosome pellet was kept. The pellet was resuspended in 1 × PBS and washed using ultracentrifugation at 100,000 g at 4 °C for an additional 120 min. The exosomes were suspended in 100 µL of 1 × PBS and kept at −80 °C for future use.

Isolated exosomes were placed on TEM grids coated with formvar/carbon and subjected to glow discharge. Afterward, the samples were stained using 2% uranyl acetate solution and analyzed using a transmission electron microscope (TEM) set at 80 kV (Titan Themis 60–300 Cubed, Thermo Fisher Scientific, USA). Dynamic light scattering (DLS, Zetasizer ZS90, Malvern Instruments Ltd., UK) was used to examine the hydrodynamic size and morphology of isolated Exos.

The presence of exosomal marker protein CD63 was revealed through western blot analysis. CD63 was isolated using RIPA buffer and its concentration was determined with a BCA Protein Assay Kit. After isolation, the protein was separated using SDS‐PAGE and then transferred to nitrocellulose membrane. The membrane blocked with skim milk for 2 h and then incubated for 2 h at room temperate with primary antibodies against CD63 protein, followed by 3 times washing with wash buffer. Then membrane incubated with HRP‐conjugated secondary antibodies for 45 min at room temperate. At the final point, DAB substrate kit used for the chromogenic detection of CD63 protein.

### 2.4 Hydrogel Preparation and Characterization

500 mg of CS was dissolved in 12 mL of 1% acetic acid solution and magnetic stirred at room temperature for 4 h. A 40% w/v solution of PEG was added and briefly agitated, then a 100 µg mL^−1^ Exos solution was introduced to achieve uniform mixing. The reaction was catalyzed by adding 30 µL of paraformaldehyde and left to run for 24 h at room temperature. Sodium bicarbonate was then added to the solution to adjust the pH to 7.2, replicating the physiological conditions. A 200 µL hydrogel solution was dispensed into a 24‐well plate and allowed to solidify at 25 °C overnight. The stable hydrogel structure formed due to electrostatic interactions between CS's positive NH2 groups and PEG's negative OH groups, as well as hydrogen bonding within the CS polymer chains. NIH/3T3 cells were seeded onto the hydrogel at a density of 2 × 10^4^ cells cm^−2^ and grown in full media. Cell morphology was observed for 72 h.

The hydrogel's structural features were examined using Fourier Transform Infrared Spectroscopy (FTIR) with a Vertex 70v vacuum spectrometer (Bruker, Germany) equipped with an Attenuated Total Reflectance (ATR) module spectroscopy in the range of 4000 and 500 cm^−1^. The gelation process was evaluated by measuring the storage (G′) and loss (G″) moduli of the hydrogel at different time points using a Discovery Hybrid Rheometer‐2 (TA Instruments, USA).

To quantify the extent of swelling, a small fragment from each group of samples was submerged in an adequate quantity of PBS (pH 7.4, T = 25 °C) until the samples attained their equilibrium weight. The samples' initial weight (W_i_) was recorded before immersion. At regular intervals, the samples were extracted from the PBS, their surfaces were dried using filter paper, and their wet weight (W_w_) was promptly measured. The average value of each swelling was calculated based on three parallel experiments. The calculation of water absorption by the films was determined using Equation (1).

(1) $$\mathrm{Swelling}\;\%\;=\left(\frac{W_{w}-W_{i}}{W_{i}}\right)\times 100$$

To examine the degradation and reduction in weight of the prepared samples, the initial weight of the dried samples (W_1_) was measured and documented. Subsequently, the samples were immersed in PBS (pH 7.4) at a temperature of 37 °C for a predetermined period of time. Following each time interval, the samples were thoroughly dehydrated, and their mass (W_2_) was documented. The degradation value was obtained by calculating the average of three parallel experiments. Equation (2) was used to calculate the percentage of degradation at specific time intervals.

(2) $$\mathrm{Degradation}\;\%\;=\left(\frac{W_{1}-W_{2}}{W_{1}}\right)\;\;\times 100$$

where W_1_ and W_2_ are the initial weight and dry weight of the samples, respectively.

The cross‐linking density (mol/cm^3^) of the hydrogels was calculated using the Flory–Rehner equation, as previously demonstrated in,^[^ ^13^ ^]^ which relates the equilibrium swelling ratio of a hydrogel to its cross‐linking density. The calculations were based on the following steps:

1. The hydrogels were immersed in deionized water at room temperature for 48 h to allow equilibrium swelling. The swollen weight (*W_s_*) and the dry weight (*W_d_*) were recorded, and the equilibrium swelling ratio was determined using Equation (3):
(3) $$Q=\frac{W_{s}}{W_{d}}\;$$
2. The polymer volume fraction (*V_r_*) in the swollen hydrogel was calculated as using Equation (4):
(4) $$V_{r}=\frac{1}{1+Q}\;$$
3. The cross‐linking density (Ve) was determined using the Flory–Rehner Equation (5):
(5) $$V_{e}=\frac{-\ln\left(1-V_{r}\right)+V_{r}+X_{1}V_{r}^{2}}{{V_{r}}^{1/3}\;V_{s}\;}\;$$

where V_r_ is polymer volume fraction in the swollen hydrogel. X_1_ is the polymer‐solvent interaction parameter. This value was assumed to be 0.55 for the CS‐PEG hydrogel, consistent with reported values for hydrophilic polymer systems in water. This value represents the average hydrophilic behavior of CS (X_1_ ≈ 0.50‐0.60) and PEG (X_1_ ≈ 0.45‐0.55) as found in the literature,^[^ ^14^, ^15^ ^]^ This approximation is commonly used in studies applying the Flory–Rehner equation to hydrogels. V_s_ is Molar volume of water, 18 cm^3^ mol^−1^.

### 2.5 Exo Loading in Hydrogels, Identification, and Release

The Exos were incorporated into the CS‐PEG hydrogel to form the Exo‐loaded hydrogel. To visually represent the dispersion of Exos within the hydrogel, they were labeled with the fluorescent dye 1,1′‐dioctadecyl‐3,3,3′,3′‐tetramethylindocarbocyanine perchlorate (DiI). In brief, a solution of DiI dye with a final concentration of 0.06 v v^−1^ was introduced to the Exo suspension. The mixture was then incubated for 20 min. An ultra‐centrifugation process was conducted at a speed of 100000 g for 90 min at a temperature of 4 °C to eliminate any non‐labeled dye. The Exo pellets labeled with DiI were suspended again in 100 µL of PBS and stored at ‐80 °C.The labeled Exos were completely mixed with hydrogel and placed in a confocal dish. The distribution of Exos was subsequently examined with Leica TCS SP8 X confocal laser scanning microscope (Leica Microsystems, Germany), and 3D images of the samples were constructed to verify the even distribution of exosomes within the hydrogel matrix.

The release kinetics of Exos from CS‐PEG ink were evaluated using a dynamic dialysis method. A dialysis membrane with a molecular weight cutoff ranging from 8000 to 12000 was used. A volume of 3 ml of the ink was inserted into a dialysis bag. The bag was then immersed in a solution of 4 ml of PBS at a pH of 6.5 and kept at a constant temperature of 35 ± 0.5 °C. Periodically, following the collection of a 1 ml sample, an equivalent amount of preheated dissolution medium was added. The quantification of the released Exos was performed using a micro BCA protein assay kit.

### 2.6 The Cytotoxicity of the Exo‐Loaded Hydrogel

An MTT experiment was performed to evaluate the effect of ADMSC‐Exos in the CS and CS‐PEG hydrogel on the proliferation of NIH/3T3 cells. The hydrogels were prepared and used to monitor changes in cell morphology during a 72‐h culture period, with media changes occurring at specific intervals (1, 3, and 5 days). Cells attached to the hydrogels were treated with 3‐(4,5‐dimethylthiazolyl‐2)‐2,5‐diphenyltetrazolium bromide (MTT; 5 mg mL^−1^ in PBS, Sigma) and then washed with PBS. The purple MTT formazan precipitates were dissolved by adding DMSO at room temperature. After a 30 min incubation, the absorbance of the samples was measured at 570 nm. Gel discs were created and placed in 24‐well plates for the microscopic observations. Each gel disk was injected with 35000 NIH/3T3 cells in 400 µL of growth media. Cell morphology was evaluated using a phase‐contrast microscope (Nikon Eclipse TS100, Nikon, Japan) on days 1, 3, and 5 of culture. Additionally, a detailed examination was performed with a Tescan Mira II scanning electron microscope (SEM) after 3 days of culture.

### 2.7 Cellular Uptake of ADMSCs‐Exo Assay

For the Exo uptake investigation, ADMSC‐Exos were labeled with DiI dye and then exposed to a 20‐min incubation at 37 °C. A control group without Exos was used as the negative control. Approximately 8000 NIH/3T3 cells were grown in a dish suitable for confocal imaging. They were then treated with DiI‐labeled Exos for 24 h at 37 °C and 5% CO_2_. The cells were fixed with 4% paraformaldehyde for 20 min after exposure. Subsequently, they were stained with a green fluorescent dye that targets F‐actin, following the supplier's guidelines. DAPI was used for nuclear staining. Leica TCS SP8 confocal laser scanning microscope was used to examine the fluorescence signals.

### 2.8 Scratch Wound Migration Assay and In Vitro Angiogenesis Measurement

The cells were then incubated at 37 °C with 5% CO_2_ to facilitate cell attachment and the formation of a dense monolayer. After reaching confluency, a sterile 200 µL pipette tip was used to make an incision in the monolayer. Subsequently, the culture media was removed, and the cells were washed with PBS. A new medium was then introduced, containing Exos at doses of 100 µg mL^−1^. The scrape was imaged 24 h after the treatment with a Leica TCS SP8 confocal laser scanning microscope. Each dish was analyzed in three specific places using MATLAB software based on a previous study,^[^ ^16^ ^]^ and each scratch test was performed three times.

HUVECs were utilized to conduct in vitro angiogenesis assays. Briefly, 50 µl of gel was added to a confocal dish and left to solidify for 45 min at a temperature of 37 °C and a CO_2_ concentration of 5%. Next, HUVECs that had been stained with CellTracker Green CMFDA and had undergone passages two to three, were placed into the dishes at a density of 1 × 10^4^ cells. Tube formation was observed after 24 h of culture using Leica TCS SP8 confocal laser scanning microscope. Image processing was performed using ImageJ software, which included measuring tube lengths and widths, as well as counting loops, where a “loop” refers to the complete circular structures formed by the tubes.

### 2.9 Mitochondrial Morphology Assessment and Reactive Oxygen Species (ROS) Level Measurement

In order to evaluate the morphology of mitochondria, NIH/3T3 cells were placed on a confocal dish. Once the cell culture reached full coverage, a sterile pipette tip with a volume of 200 µL was employed to create an incision in the monolayer. The culture medium was extracted and the cells were rinsed with PBS. The Mitolite Red FX600 solution was introduced and allowed to incubate for 30 min at 37 °C. The cellular mitochondrial morphology in the vicinity of the wound (in the wound state) and at a distance from the wound (in the normal state) was examined using a Leica TCS SP8 confocal laser scanning microscope.

The quantification of cellular ROS generation was conducted with alterations following the previously established protocol. Once the NIH/3T3 cells had completely covered the surface, a clean 200 µL pipette tip was used to cut the layer of cells. The nutrient solution was extracted and the cells were rinsed with PBS. The cells were subsequently treated with MitoSOX Red (5 µM) and incubated for 10 min at 37 °C. Leica TCS SP8 confocal laser scanning microscope was used to document the red fluorescence, which indicates levels of ROS. Quantitative analysis of mean fluorescence level was performed in MATLAB software. Grayscale microscopic images were binarized by plain thresholding and processed using morphological filters to separate cell and cell‐free areas and create a mask. Then, the fluorescence intensity level was determined using the mask by averaging the pixel intensities only in cell areas.

### 2.10 Gene Expression Analysis

The expression levels of VEGF and NF‐κB genes in HUVECs cultured on the samples were measured using qPCR on day 10. The RNA was extracted using the RNeasy Mini Kit and its concentration was determined using a NanoPhotometer (IMPLEN, Munich, Germany). The RevertAid First Strand cDNA Synthesis Kit was used to synthesize cDNA. The VEGF and NF‐κB genes were measured using the LightCycler 480 High Resolution Melting Master SYBR Green amplification kit. The measurements were conducted on the croBEE RT‐PCR System (GeneProof, Brno, Czech Republic). The primers used for VEGF were GGCGCTACCTGTATCAATGG (forward) and TCAGCCAACTCGTCACAGTC (reverse). The primers used for NF‐κB were GGCGCTACCTGTATCAATGG (forward) and TCAGCCAACTCGTCACAGTC (reverse). The PCR protocol consisted of an initial denaturation step at 95 °C for 5 min, followed by 35 cycles of amplification at 95 °C for 30 s, 58 °C for 30 s, and 72 °C for 30 s. The housekeeping gene used for all experiments was GAPDH, with the forward primer sequence GGTCGGAGTCAACGGATTTG and the reverse primer sequence ATGAGCCCCAGCCTTCTCCAT. The gene expression was quantified relative to a reference using the ΔΔCt method. Three samples (n = 3) were examined from each treatment group.

### 2.11 Statistical Analysis

The experiments were conducted in triplicate, and results are presented as mean ± standard error of the mean. Group comparisons were performed using one‐way analysis of variance (ANOVA), with statistical significance set at *p < 0.05.

## 3 Results

### 3.1 Exo Characterization

**Figure 1:**
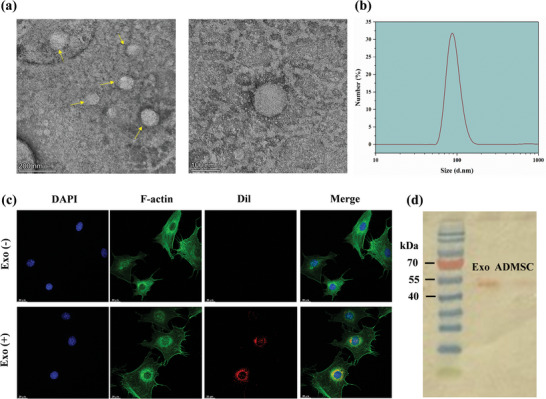
Exo characterization analyses. a) TEM images of isolated Exos showing their typical bilayer membrane structure with an average diameter of ≈100 nm. Scale bars: 200 and 100 nm. b) Size distribution of Exos measured by DLS. c) Confocal microscopy images showing the uptake of DiI‐labeled Exos by NIH/3T3 cells. DAPI (blue) stains the nuclei, F‐actin (green) stains the cytoskeleton, and DiI (red) marks the Exos. The merged images confirm the presence of Exos within the cytoplasm of NIH/3T3 cells. d) Western blot analysis demonstrating the presence of the exosomal marker CD63 in the isolated Exos from ADMSCs.

TEM examination showed that the separated Exos had an average diameter of ≈ 100 nm and displayed a bilayer membrane structure (**Figure** **1**a). The size distribution of the isolated Exos was determined using DLS. The findings, depicted in Figure 1b demonstrate a limited range of sizes with a prominent concentration ≈ 100 nm. This indicates a uniform population of Exos, which aligns with the usual size range of MSCs‐Exos. The Exos were effectively marked with the fluorescent dye DiI, making it easier to observe them (Figure 1c). When introduced to NIH/3T3 fibroblast cells, the DiI‐labeled Exos were mainly taken up into the cytoplasm, with a small portion also seen in the nuclear regions. This experiment indicates that Exos can be taken up by recipient cells, potentially triggering their biological activities. Western blot examination verified the existence of CD63 in the Exos, a recognized exosomal marker (Figure 1d), and the cell lysate conditions which is corresponded to expression of CD63.

### 3.2 Effect of Exo on Migration and Angiogenesis

**Figure 2:**
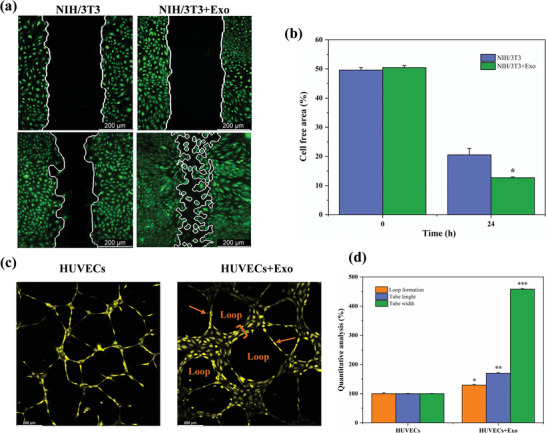
Evaluation of Exos effects on NIH/3T3 cell migration and HUVECs tube formation: a) Representative confocal images of scratch wound healing assay for NIH/3T3 cells treated with or without Exo after 24 h. The white outlines indicate the cell‐free areas at the beginning (0 h) and after 24 h. b) Quantitative analysis of the percentage of cell‐free area in NIH/3T3 cultures at 0 and 24 h. c) Representative images of tube formation assay for HUVECs treated with or without exosomes. The arrows point to newly formed tubular structures, while the term “Loop” indicates the complete circular structures formed by the tubes. d) Quantitative analysis of loop formation, tube length, and tube width in the tube formation assay. Exos treatment significantly increased all measured parameters, demonstrating the pro‐angiogenic effect of Exos (*p < 0.05, **p < 0.01, ***p < 0.001).

The influence of Exos on the migration of NIH/3T3 cells was evaluated using a scratch wound assay. Over 24 h, the migration rate of NIH/3T3 cells treated with Exos was significantly increased compared to the untreated control group. **Figure** **2**a demonstrates that after 24 h of incubation, the control group (NIH/3T3) displayed a cell‐free area, indicating that wound closure was not fully achieved. On the other hand, the group that received Exos (NIH/3T3+Exo) showed a greater decrease in the area without cells, indicating faster movement of cells. The quantitative analysis provided additional evidence that the group treated with exosomes achieved a significantly greater degree of wound closure. The cell‐free area was reduced to ≈10% in the exosome‐treated group, compared to 20% in the control group (*p* < 0.05), as depicted in Figure 2b.

Furthermore, the Exos not only improved cell migration but also stimulated angiogenesis. The tube formation assay, as depicted in Figure 2c, revealed that HUVECs cultured with Exos (HUVECs+Exo) exhibited enhanced and enclosed vascular networks, characterized by a higher number of loops and thicker, longer tubes, in comparison to the HUVECs cultured without Exos. The quantitative analysis in Figure 2d demonstrates a substantial increase in the formation of loops, tube length, and tube width in the exosome‐treated group.

### 3.3 Mitochondrial Morphology and ROS Level Assessment

**Figure 3:**
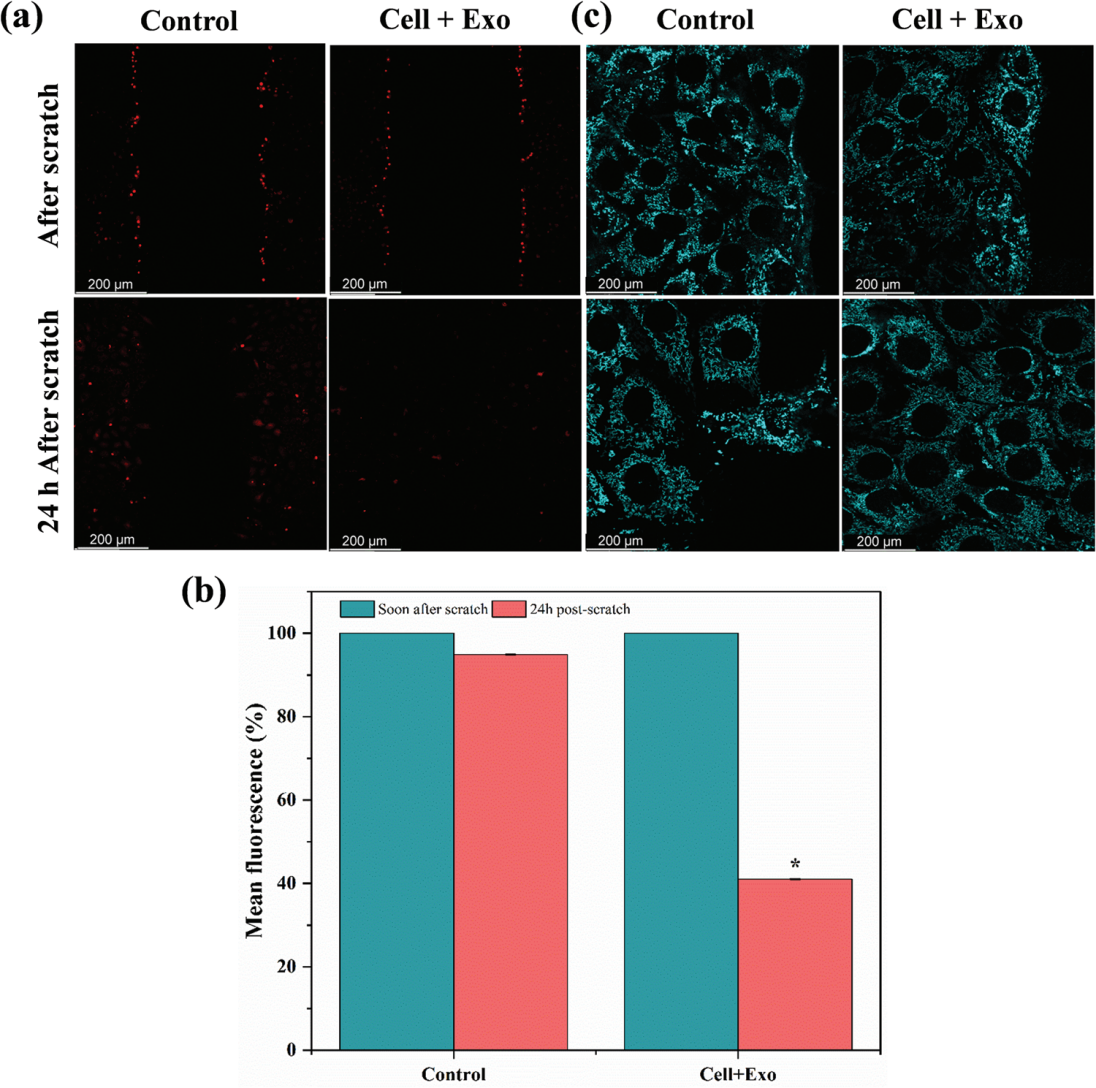
Evaluation of ROS levels and mitochondrial structure in NIH/3T3 cells after scratch injury and treatment with Exos. a) Representative confocal images showing ROS levels (red fluorescence) in control and Exo‐treated groups immediately after the scratch and 24 h post‐scratch. b) Quantitative analysis of mean fluorescence intensity (%) for ROS levels soon after the scratch and 24 h post‐scratch, demonstrating a significant reduction in ROS in the Exo‐treated group (*p < 0.05). c) Mitochondrial structure visualized by fluorescence microscopy in NIH/3T3 cells near the scratch area, showing compromised mitochondria in the control group and improved mitochondrial integrity in the Exo‐treated group.

Oxidative stress in the wound area was evaluated by measuring the level of ROS after 24 h (**Figure** **3**a). The levels of ROS were significantly elevated in the control samples (samples without Exos), suggesting an increase in oxidative stress. Nevertheless, the incorporation of Exos resulted in a notable decrease in ROS levels in the vicinity of the wound site, as indicated by the quantitative analysis of mean fluorescence in Figure 3b.

The mitochondrial structure of NIH/3T3 cells near the scratch area was found to be compromised in the control samples (without Exos) after 24 h of culture. The mitochondria exhibited signs of fragmentation and dysfunction, as depicted in Figure 3c. On the other hand, the samples that were exposed to Exos exhibited a notable enhancement in the structure of their mitochondria near the injured region. These mitochondria appeared elongated and in a healthier state compared to the group that did not receive the Exos.

### 3.4 CS‐PEG Hydrogel Characterization

**Figure 4:**
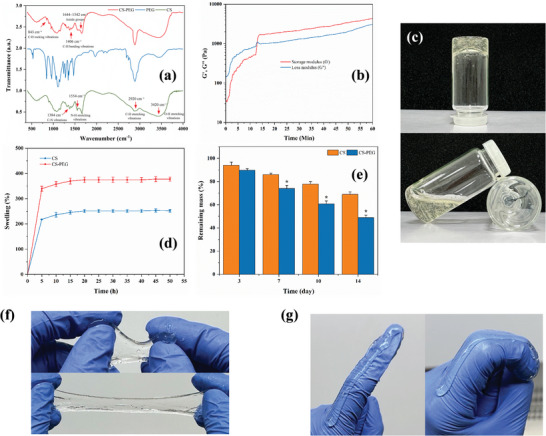
Characterization of CS‐PEG Hydrogel. a) FTIR spectra of CS‐PEG hydrogel, PEG, and chitosan, displaying the chemical bonds and functional groups present. b) Rheological analysis showing the storage modulus (G') and loss modulus (G””) over time, indicating the gelation process of the hydrogel. c) Visual demonstration of the physical appearance and integrity of the CS‐PEG hydrogel in vials, illustrating its structural stability. d) Swelling behavior of CS‐PEG hydrogel compared to pure chitosan over time, showing the hydrogel's capacity to absorb water. e) Degradation profile of the hydrogels over 14 days, highlighting the sustained mass of CS‐PEG hydrogel. Data are presented as mean ± SD; n = 3. *p < 0.05 compared to CS. f,g) Representative images of the CS‐PEG hydrogel demonstrating its flexibility, stretchability, and adaptability as a wound dressing material.

The chemical composition of the CS‐PEG hydrogel was examined through FTIR spectroscopy as shown in **Figure** **4**a, which unveiled a range of chemical bonds. The CS spectra exhibited a peak at 3420 cm⁻¹, corresponding to O─H stretching vibrations, which are common in both chitosan and PEG due to the presence of hydroxyl groups. The spectral range between 3150–3600 cm^−1^ is commonly linked to the vibrations of O─H and NH─ bonds during stretching. The presence of a peak at 2920 cm^−1^ suggests the occurrence of C─H stretching in CS.^[^ ^17^ ^]^ Additionally, the peak at 1554 cm^−1^ indicates the presence of N─H bending vibrations in CS. The peak observed at 1384 cm^−1^ was attributed to C–N vibrations, while the range from 1150–1021 cm^−1^ indicated the presence of antisymmetric stretching of C─O─C and C─O group stretching vibrations.^[^ ^18^ ^]^ The addition of PEG to the CS matrix was confirmed by the broad peak around 3294 cm⁻¹, which is indicative of O─H stretching vibrations.^[^ ^19^ ^]^ This peak is characteristic of both CS and PEG, reflecting the presence of hydroxyl groups in the hydrogel matrix. Furthermore, there were additional peaks observed between 1644–1542 cm^−1^, suggesting the existence of amide groups. The peak at 1406 cm⁻¹ is associated with C─H bending vibrations, which may indicate the presence of ─CH₃ groups. The characteristic peaks of PEG are identified at 1280 cm⁻¹, corresponding to C─O─C stretching vibrations, and at 843 cm⁻¹, which is linked to C─H rocking vibrations,^[^ ^20^ ^]^ confirming the incorporation of PEG into the composite. The rheological measurements (Figure 4b) provided confirmation of the gelation process of the CS‐PEG hydrogel. The storage modulus (G′) surpassed the loss modulus (G′′) ≈ 10 min, indicating the initiation of gel state transformation and confirming the successful establishment of a stable hydrogel network. The gelation process of the CS‐PEG hydrogel was also observed by turning the tube upside down (Figure 4c).

The hydrogels were assessed for their swelling behavior over a period of time and the results are presented in Figure 4d. The CS‐PEG hydrogel demonstrated a greater degree of swelling in comparison to the pure CS hydrogel, achieving equilibrium after ≈48 h. The hydrogel's high‐water absorption capacity is advantageous for wound healing as it helps to maintain a moist environment at the wound site. To further evaluate the structural properties of the hydrogels, the cross‐linking density was calculated using the Flory–Rehner equation based on the equilibrium swelling ratio. The results revealed that the cross‐linking density of the CS‐PEG hydrogel was 0.0435 mol cm^−^^3^, compared to 0.0563 mol cm^−^^3^ for the pure CS hydrogel. The reduction in cross‐linking density upon PEG incorporation can be attributed to the disruption of rigid chitosan chains by PEG, which introduces flexibility and reduces the number of effective cross‐links per unit volume. This is consistent with the observed swelling behavior, where the CS‐PEG hydrogel exhibited a higher equilibrium swelling ratio (377.56%) than the pure CS hydrogel (252.2%). These results suggest that PEG incorporation improves the balance between elasticity and stability, making the CS‐PEG hydrogel more suitable for applications requiring prolonged retention and controlled degradation. The hydrogels' degradation profile was observed for a duration of 14 days (Figure 4e). The CS‐PEG hydrogel exhibited a regulated degradation rate, experiencing a substantial decrease in mass over a period of time, while still maintaining ≈40% of its original mass after 14 days. This showcases its capacity for long‐term therapeutic administration and structural reinforcement throughout the wound healing process. The images of the CS‐PEG hydrogel (Figure 4f,g) demonstrate its flexibility and stretchability, emphasizing its appropriateness for application as a wound dressing. The images demonstrate the gel's capacity to be manipulated without fracturing, indicating excellent mechanical characteristics. The hydrogel's flexibility allows it to adapt to the wound site, creating a barrier that protects while allowing for movement.

### 3.5 Exos Distribution in the CS‐PEG Hydrogel and Release Profile

**Figure 5:**
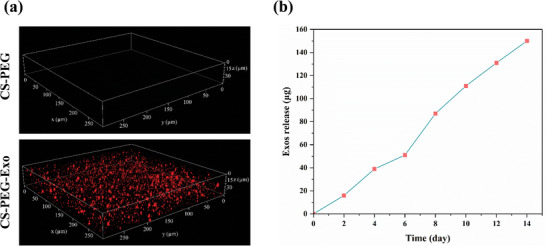
a) Confocal microscopy 3D reconstruction images of the CS‐PEG hydrogel showing the distribution of Exos within the hydrogel matrix. The top image represents the hydrogel before Exo incorporation, and the bottom image shows the hydrogel after successful incorporation, with red fluorescence indicating the presence of Exos. b) Cumulative release profile of Exos from the chitosan‐PEG hydrogel over 14 days.

The successful integration of Exos into the chitosan‐PEG hydrogel matrix was verified through confocal microscopy, as depicted in **Figure** **5**a. The 3D reconstruction images depict the spatial arrangement of Exos within the hydrogel structure. The upper image depicts the hydrogel before the inclusion of Exo. In contrast, the lower image exhibits the hydrogel containing Exos, as indicated by the dense red fluorescence signal evenly distributed throughout the matrix. This distribution verifies the successful integration of the Exos into the hydrogel, which is essential for maintaining a continuous therapeutic release.

Figure 5b illustrates the release profile of Exos from the hydrogel. The graph illustrates the progressive discharge of Exos over 14 days, demonstrating a consistent and regulated release of Exos from the hydrogel matrix. The quantity of Exos released steadily increased over time, reaching an approximate value of 140 µg by day 14. The consistent and continuous release of the hydrogel indicates that it can deliver long‐lasting therapeutic effects, promoting the healing of wounds for an extended duration.

### 3.6 Cell Viability

**Figure 6:**
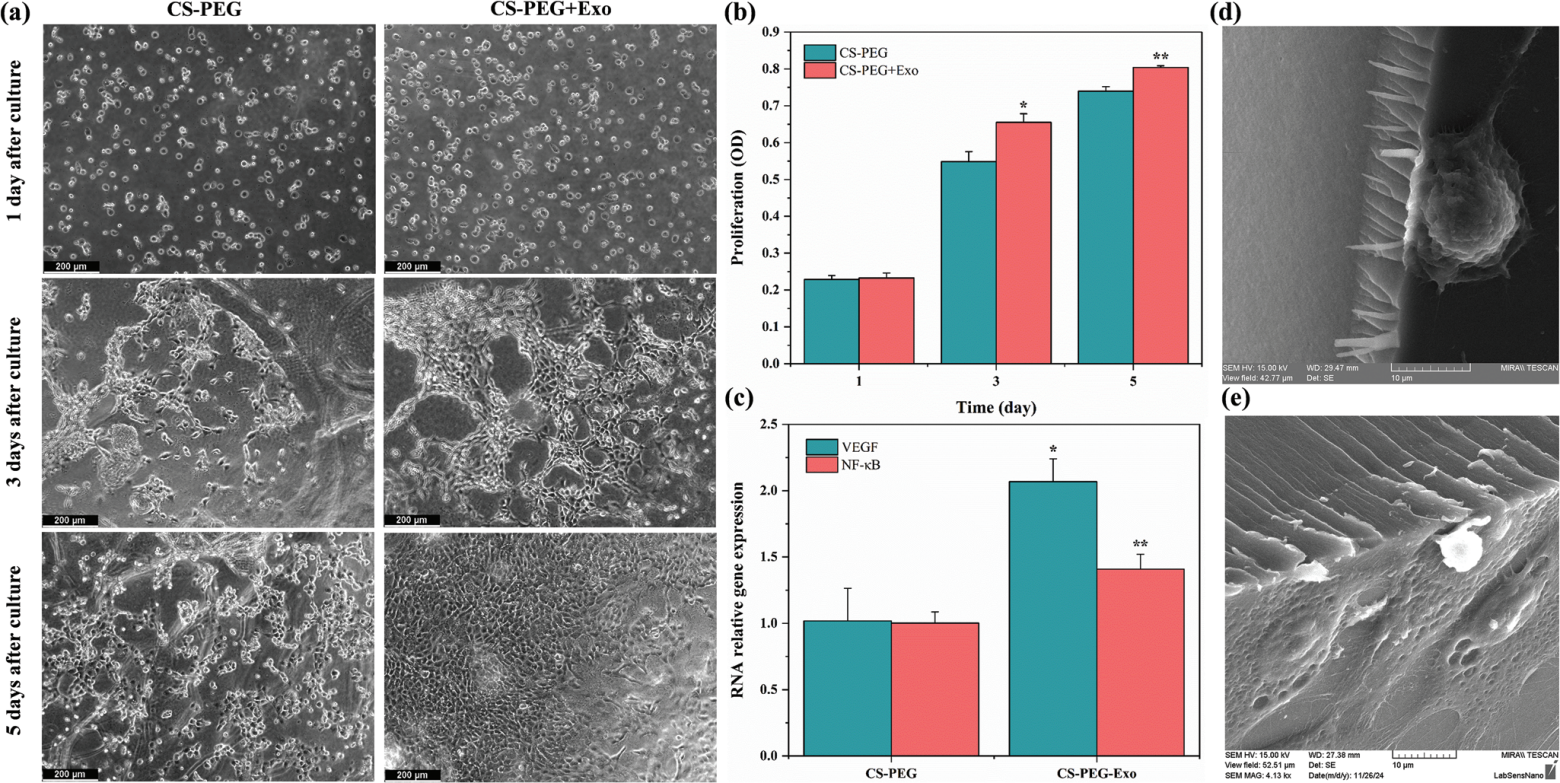
a) Morphological observation of NIH/3T3 cells cultured on CS‐PEG and CS‐PEG‐Exo hydrogels over a period of 5 days. b) Quantitative analysis of cell proliferation on CS‐PEG and CS‐PEG‐Exo hydrogels measured by MTT assay at 1, 3, and 5 days. CS‐PEG‐Exo hydrogel shows significantly higher proliferation rates compared to CS‐PEG hydrogel (*p < 0.05, **p < 0.01). c) Relative gene expression levels of VEGF and NF‐κB in HUVECs cultured on CS‐PEG and CS‐PEG‐Exo hydrogels. VEGF expression is notably higher in the CS‐PEG‐Exo group, indicating enhanced angiogenesis (*p < 0.05). NF‐κB expression is also significantly elevated in the CS‐PEG‐Exo group, suggesting improved inflammatory response regulation (**p < 0.01). d) SEM images of NIH/3T3 cells cultured on the surface of CS‐PEG hydrogel without Exos show limited cellular interaction, with minimal adhesion or spreading, and on e) CS‐PEG hydrogel with Exos demonstrates excellent cell adhesion and spreading, with visible cellular extensions (filopodia), indicating enhanced biocompatibility.

NIH/3T3 cells were cultured directly on CS‐PEG hydrogel substrates to assess their biocompatibility and the efficacy of their combination with Exos. **Figure** **6**a depicts the cellular morphology and structural characteristics of CS‐PEG and CS‐PEG‐Exo hydrogels for a duration of 5 days. Within 24 h, the cells exhibited optimal spreading on the surface of the CS‐PEG‐Exo hydrogel, resembling the typical conditions observed during subculture. Cell proliferation and expansion exhibited a steady and continuous enhancement throughout the 5‐day incubation duration. On the other hand, cells grown on CS‐PEG hydrogels stuck to and grew in a round shape, continuing to multiply in groups, although their rate of growth was slower compared to other conditions. The quantification of cell proliferation on different substrates was performed using the MTT test, as depicted in Figure 6b. The number of cells on the CS‐PEG‐Exo hydrogel was significantly greater than on the CS‐PEG hydrogel at 3, and 5 days after seeding with the most significant increase observed at day 5 (*p < 0.05 and **p < 0.01). To further evaluate the interaction between cells and the hydrogel during in vitro culture, SEM analysis was conducted after 3 days of NIH/3T3 cell culture on the hydrogel surface. As shown in Figure 6d,e, SEM images revealed well‐spread NIH/3T3 cells adhered to the hydrogel surface, particularly on the Exos‐loaded hydrogels. The cells exhibited intact and healthy morphology, confirming the hydrogel's biocompatibility and ability to support cellular adhesion and proliferation. While significant infiltration into the hydrogel matrix was not observed, the surface interactions demonstrate the hydrogel's ability to promote cellular activity.

Additionally, the hydrogel maintained its structural integrity and hydration throughout the culture period, as evidenced by its stable surface morphology (Figure 6d,e), swelling behavior (Figure 4d), and degradation profile (Figure 4e). These properties ensure the hydrogel provides a moist and stable environment, critical for cell survival and proliferation during wound healing. Together, these findings demonstrate that the CS‐PEG‐Exo hydrogel is capable of supporting cell attachment and promoting cellular activity while maintaining its structural stability.

### 3.7 Gene Expression Analysis

Figure 6c illustrates the comparative levels of expression of VEGF and NF‐κB genes in HUVECs cultured on the hydrogel samples. The expression of VEGF in the CS‐PEG‐Exo group was significantly higher, approximately double that of the CS‐PEG group (*p < 0.05), suggesting an increased potential for angiogenesis. In a similar manner, the expression level of NF‐κB in HUVECs cultured on the CS‐PEG‐Exo hydrogel was significantly increased compared to the CS‐PEG group (**p < 0.01). This indicates that the inclusion of Exos not only enhances angiogenesis but also regulates the inflammatory response.

## 4 Discussion

Efficient wound healing continues to be a notable clinical obstacle, especially in achieving prompt recovery and minimizing scar formation. Conventional approaches frequently fall short in maximizing the intricate biological mechanisms necessary for effective tissue regeneration.^[^ ^21^ ^]^ This study aims to overcome these difficulties by creating a chitosan‐PEG hydrogel that contains Exos. The purpose of this hydrogel is to improve wound healing by specifically targeting cellular and molecular processes. This innovative dressing utilizes the anti‐inflammatory and regenerative characteristics of Exos to enhance key elements of wound healing, such as cell migration, the formation of new blood vessels, and the protection of mitochondria, ultimately facilitating faster and more efficient tissue repair.

MSCs‐Exos have garnered interest due to their anti‐inflammatory and immunomodulatory characteristics, which are comparable to those of MSCs. The objective of this study was to develop a CS‐PEG‐Exos hydrogel dressing that can promote wound healing through the sustained release of Exos. The successful identification and analysis of Exos were verified using TEM, the presence of marker proteins, and DLS. The distribution profile may verify that the isolation technique was successful in generating exosomes with consistent size, which is crucial for ensuring reliability and uniformity in therapeutic uses. The successful incorporation of these Exos into NIH/3T3 cells demonstrates their ability to facilitate communication between cells and their potential for therapeutic effectiveness.

CS‐based hydrogels are considered to be highly favorable for use as wound dressings due to their biocompatibility, biodegradability, and antimicrobial properties.^[^ ^22^ ^]^ The addition of PEG to CS can improve the mechanical properties, flexibility, and controlled breakdown of the hydrogel.^[^ ^23^ ^]^ This enhancement allows for the sustained release of Exos, which is important for achieving long‐lasting therapeutic effects in wound healing. The confirmation of a stable hydrogel network, as evidenced by FTIR spectroscopy and rheological analysis, emphasizes the durability and medical applicability of this composite material. The successful incorporation of PEG into the chitosan matrix is confirmed by the identification of important functional groups in the FTIR spectra. This integration greatly improves the mechanical properties and durability of the hydrogel. These enhancements are essential to ensure that the dressing adheres effectively to wound sites, offering consistent protection and long‐lasting therapeutic effects. The rheological characteristics of the CS‐PEG‐Exos hydrogel, specifically its gelation process, indicate its capacity to create a durable and resilient protective layer on wound surfaces. Ensuring the hydrogel's structural integrity is crucial for effective and prolonged Exo delivery during application. The addition of PEG to the hydrogel enhances its viscoelastic properties, creating a harmonious balance between rigidity and elasticity. This balance is crucial for effectively using the hydrogel on dynamic wound sites. The balance of properties in the hydrogel enables it to maintain flexibility while still providing protection, which makes it well‐suited for use in areas that experience frequent movement.

The hydrogel's controlled swelling behavior additionally enhances its efficacy in wound management by sustaining a humid environment, which expedites the healing process and diminishes scarring.^[^ ^24^ ^]^ PEG enhances the hydrogel's ability to absorb water, which is crucial for maintaining moisture at the wound location.^[^ ^25^ ^]^ The degradation characteristics of the hydrogel demonstrate its capacity to deliver sustained therapeutic effects, facilitated by the presence of PEG which promotes a consistent and regulated degradation process, enabling the continuous release of Exos for 14 days. The extended duration of this presence at the wound site facilitates essential processes such as the regulation of inflammation, angiogenesis, and the cellular migration.^[^ ^26^ ^]^ Moreover, the hydrogel's flexibility and elasticity offer practical benefits when used as a wound dressing. It can easily adapt to different wound shapes and retain its structural integrity even during physical activity. This ensures reliable therapeutic effects and minimizes the likelihood of discomfort or additional harm.^[^ ^27^ ^]^

The migration assays revealed that the CS‐PEG‐Exo hydrogel substantially enhanced the migration of NIH/3T3 cells in comparison to the control group. The increased ability of cells to move is essential for the process of wound healing, as it enables cells to migrate into the area of the wound, aiding in its closure. In addition, the angiogenesis study demonstrated that the hydrogel incorporating Exo successfully promoted the development of blood vessels. This phenomenon is likely driven by the presence of growth factors and signaling molecules within the Exos.^[^ ^28^ ^]^ These bioactive substances stimulate specific signaling pathways that are crucial for cell migration and angiogenesis, thus facilitating tissue repair.^[^ ^29^ ^]^ Angiogenesis is crucial because it facilitates the delivery of oxygen and nutrients to the healing tissues, which is essential for efficient and prompt wound healing.^[^ ^30^ ^]^ The results of our research align with previous investigations, which have shown that Exo has a crucial function in facilitating the growth of new blood vessels and the healing of wounds by transporting biologically active substances such as growth factors, cytokines, and microRNAs,^[^ ^31^, ^32^ ^]^

Our results revealed that ADMSCs‐Exos aid in maintaining the integrity and functionality of mitochondria, which is essential for cellular energy generation and overall cellular health. The CS‐PEG‐Exo hydrogel exhibited a protective impact on mitochondria in NIH/3T3 cells, with preserving their elongated and undamaged structure, in contrast to the fragmented mitochondria observed in the control group. The ability to defend against oxidative stress, as indicated by lower levels of ROS, is crucial for successful wound healing.^[^ ^33^ ^]^ Exos aid in reducing oxidative stress, which has the potential to harm cellular constituents and stimulate inflammation.^[^ ^34^ ^]^ In addition, previous studies have shown that Exos seem to control antioxidant enzymes and reduce pro‐inflammatory cytokines, providing further evidence of their ability to decrease oxidative stress and inflammation.^[^ ^35^ ^]^

Our findings were further supported by the analysis of gene expression. It was found that Exo substantially increase the expression of VEGF in HUVECs cultured on the CS‐PEG‐Exo hydrogel. This indicates an improved ability for angiogenesis. VEGF is a crucial factor that promotes the formation of new blood vessels by stimulating the growth, migration, and specialization of endothelial cells. This process is necessary for both normal tissue repair and in diseases such as tumor development.^[^ ^36^ ^]^ Exos have demonstrated the ability to enhance the expression of VEGF in endothelial cells, thereby promoting the process of angiogenesis.^[^ ^37^ ^]^ In addition, our study noted a rise in NF‐κB expression in HUVECs cultured on the CS‐PEG‐Exo hydrogel. The NF‐κB pathway has a crucial function in controlling immune responses and inflammation.^[^ ^38^ ^]^ Additionally, it can stimulate the production of VEGF in endothelial cells, leading to a self‐amplifying process that boosts angiogenesis.^[^ ^39^ ^]^ The activation of this pathway by Exo may demonstrate their ability to both promote angiogenesis and regulate the inflammatory response, both of which are essential for effective wound healing.^[^ ^40^ ^]^

Future research should focus on addressing the limitations of this study and expanding the potential applications of CS‐PEG‐Exo hydrogels. One limitation of this study is the lack of significant cell infiltration into the hydrogel matrix, as observed in SEM analysis after 3 days of cell culture. This limited infiltration is likely due to the hydrogel's structural and cross‐linking properties, which were optimized for stability and controlled exosome release rather than promoting deep cellular penetration. While this design aligns well with the hydrogel's primary purpose of wound healing, where surface‐level cellular adhesion, proliferation, and migration are critical, future modifications to enhance cellular penetration could broaden its applicability. For instance, tailoring the hydrogel's porosity, cross‐linking density, or degradation rate may enable better cell integration for applications requiring deep cellular migration or vascularization throughout the scaffold.

Future work should also involve in vivo studies to evaluate the hydrogel's therapeutic efficacy and biocompatibility under physiological conditions. These studies would provide insights into its long‐term performance, particularly in complex wound healing scenarios like diabetic ulcers or burns. Moreover, exploring the functionalization of the hydrogel by incorporating antimicrobial agents or additional bioactive molecules could further enhance its applicability, particularly in infected wounds or other challenging environments. Beyond wound healing, the hydrogel's‐controlled release mechanism could be leveraged for drug delivery applications, targeting conditions such as inflammatory diseases or localized cancer therapy. Additionally, tailoring the hydrogel's mechanical properties (e.g., stiffness or elasticity) to match specific tissue requirements could expand its use in regenerative medicine, including cartilage or bone tissue engineering. Addressing these directions could significantly enhance the translational potential of CS‐PEG‐Exo hydrogels for diverse medical applications.

## 5 Conclusion

In this study, we developed a hydrogel made of CS and PEG that incorporates ADMSCs‐Exo to enhance the healing of skin wounds. Our findings suggested that the inclusion of ADMSCs‐Exos in a CS‐PEG hydrogel greatly enhances its efficacy as a wound dressing. The study provides evidence that the hydrogel containing Exo has the potential to improve cell migration, promote the formation of blood vessels, maintain mitochondrial health, decrease oxidative stress, and control inflammation. The combined effects of these factors contribute to a more efficient healing of wounds and regeneration of tissues, providing a promising approach for advanced therapies in wound care. Future research should prioritize the optimization of the hydrogel formulation for clinical use and evaluate its efficacy in more intricate wound models to fully harness its regenerative capabilities.

## Conflict of Interest

The authors declare no conflict of interest.

## Data Availability

The data that support the findings of this study are available from the corresponding author upon reasonable request.
